# Microwave Foaming of Materials: An Emerging Field

**DOI:** 10.3390/polym12112477

**Published:** 2020-10-25

**Authors:** Mukarram Zubair, Rebecca Ferrari, Omar Alagha, Nuhu Dalhat Mu’azu, Nawaf I. Blaisi, Ijlal Shahrukh Ateeq, Mohammad Saood Manzar

**Affiliations:** 1 Department of Environmental Engineering, College of Engineering, Imam Abdulrahman Bin Faisal University, P.O. Box 1982, Dammam 31451, Saudi Arabia; mzzubair@iau.edu.sa (M.Z.); nmdalhat@iau.edu.sa (N.D.M.); niblaisi@iau.edu.sa (N.I.B.); msmanzar@iau.edu.sa (M.S.M.); 2 Food, Water, Waste Research Group, Faculty of Engineering, University of Nottingham, University Park, Nottingham NG7 2RD, UK; becca.ferrari@nottingham.ac.uk; 3 Department of Biomedical Engineering, College of Engineering, Imam Abdulrahman Bin Faisal University, P.O. Box 1982, Dammam 31451, Saudi Arabia; Lsateeq@iau.edu.sa

**Keywords:** foam, microwave heating, processing, polymer, biopolymer

## Abstract

In the last two decades, the application of microwave heating to the processing of materials has to become increasingly widespread. Microwave-assisted foaming processes show promise for industrial commercialization due to the potential advantages that microwaves have shown compared to conventional methods. These include reducing process time, improved energy efficiency, solvent-free foaming, reduced processing steps, and improved product quality. However, the interaction of microwave energy with foaming materials, the effects of critical processing factors on microwave foaming behavior, and the foamed product’s final properties are still not well-explored. This article reviews the mechanism and principles of microwave foaming of different materials. The article critically evaluates the impact of influential foaming parameters such as blowing agent, viscosity, precursor properties, microwave conditions, additives, and filler on the interaction of microwave, foaming material, physical (expansion, cellular structure, and density), mechanical, and thermal properties of the resultant foamed product. Finally, the key challenges and opportunities for developing industrial microwave foaming processes are identified, and areas for potential future research works are highlighted.

## 1. Introduction

Foam materials exhibit a unique combination of properties that includes low density, low thermal conductivity, excellent insulation, and mechanical properties. The previous properties render them highly attractive materials for many industrial applications. Over the last two decades, foamed materials have been adopted in applications as diverse as packaging [1], automotive, building/construction [2], insulation and furniture, sports [3,4], environmental remediation [5,6], drug delivery, and tissue engineering [7,8]. Polymeric foams are the most commonly used foam materials, and are commercially termed “plastic foams”. Bio-foams, another type of foam produced from bio-based materials, have received tremendous attention and show great industrial potential. Other materials besides these such as phenolic foams [9], metallic foams [10], and carbon foams [11] have been reported and are commercially used in the aerospace, construction [12], military [13], and supercapacitor [14] industries.

Conventionally, foamed materials are produced using processes that can be categorized as batch, injection, or extrusion processes. Process selection depends predominantly on the precursor raw materials, desired characteristics of the final product, and the target applications [15,16,17]. Batch processes are usually undertaken at a smaller scale to research and develop new materials or the study of foaming. At an industrial scale, injection and extrusion techniques are used within semi-batch and continuous processes to enable economically viable production [18,19]. Extrusion is the most widely used industrial technique for preparing polymeric foams due to its low cost [12]. However, studies have revealed that extrusion is not the most effective process for producing a well-controlled cellular structure in foams. This is due to poor porosity control; sometimes, there is a need for an additional processing step to remove accumulated solvent residues [20]. In order to overcome this issue, other foaming techniques such as modified supercritical fluids extrusion [11,14], freeze-drying/solvent exchange [9,21], and microwave-assisted foaming [22,23] have been investigated as routes to a uniform cellular structure as well as for better controlling the porosity of products. In recent decades, microwave processing has proved to be an attractive alternative heating method for materials processing. It has been effectively used for various applications from laboratory to industrial scales such as polymer processing [24], food processing [25], nanomaterial synthesis [26,27], foaming [28], pyrolysis [29], remediation of pollutants [30], and ore sorting [31]. Table 1 summarizes the main foaming methods used in the literature and their application for producing various types of foamed products.

The benefits of microwave heating arise from its ability to provide volumetric heating of the bulk of the material and selective heating to particular phases with materials [23,24]. These benefits include easy on/off heating control and reduced processing time, faster healing rates, bulk penetration, which overcomes heat transfer limitations, the potential for lower energy consumption, and improved product quality compared to conventional heating [35,36]. The resulting process savings include reduced energy costs and lower product costs from higher throughputs [37].

Microwave processing has shown promise within the foaming industry. It has mainly been adopted for the fabrication of various kinds of foams such as plastic foams [38,39], starch foams [40], phenolic foams [38], and metallic foams [41]. Previous studies on microwave foaming methods highlighted its potential as a rapid and sustainable technique with little or zero dependence on blowing agents [42,43]. Moreover, the process has a high potential for the fabrication of both high- and low-density foams [9,44], controllable and even distribution of temperature resulting in a uniform cellular structure with low thermal conductivity foams [15,45], and formation of highly porous scaffolds with excellent mechanical and thermal characteristics [43,46,47]. For instance, the compressive stress of temple and superfine flour foams produced via microwave foaming technique are 300 and 200 kPa, respectively, at 15% strain. This was relatively higher than that of the EPS cushion block (180 kPa) and a wheat flour loose-fill foam (20 kPa) [48].

Similarly, compressive strength wheat starch reinforced with straw fibers fabricated using microwave heating was 0.0065 (MPa), which is relatively better than commercial biobased foam products [49,50,51]. These starch-based foams showed excellent mechanical and thermal stability at temperature ranges of 85 °C to 95 °C [46]. Microwave foaming has successfully produced cellular phenolic foams exhibiting low thermal conductivity of 0.029 to 0.064 (W/mK) [15]. These values are comparable to commercial polyurethane (PU) foams of thermal conductivity of 0.022 and 0.035 (W/m∙K). Moreover, a high compressive strength of 1249–2170 (KPa) was also reported of phenolic foam reinforced with carbon by microwave foaming technique [52]. Recently, microwave-assisted chemical foaming and microwave hybrid heating have been reported to develop highly porous and thermally stable metallic foams that can be utilized as heat exchangers, catalyst substrates, and filters at elevated temperatures [45,53,54]. This has enabled the development of high-performance microwave-assisted foaming processes to produce materials for a diverse range of applications [55,56].

The resulting characteristics of microwave-assisted foams—such as more uniform cellular structure, low density, conductivity, and mechanical and thermal stability—depends on key influential factors, namely microwave power (or power density), properties of precursor, blowing or nucleation agent, the addition of microwave absorbers (susceptors), moisture content, viscosity, and additives (salts, fiber, glass, etc.). In order to realize the benefits of microwave processing, the fundamental mechanisms involved during microwave foaming and their key controlling factors need to be better understood.

Although microwave processing is a growing area of research, reviews in this field have focused so far on the application of microwave heating in environmental engineering [51], food processing technologies [57], and chemical synthesis [58], with very few studies that focused only on the foaming materials [59,60,61] beside microwave heating [29,62]. Explicitly, there is no systematic review article on microwaves’ application to foaming technology based on our detailed literature review. Therefore, this review article gives an in-depth description and mechanistic understanding of physical and chemical processes and steps involved during microwave foaming of different materials within the literature. The influence of critical microwave foaming parameters such as blowing agent, viscosity, microwave power, and exposure time, and reinforcing and nucleating agents on the behavior of final physical, mechanical, and thermal properties of foams are critically evaluated. Finally, the key challenges and opportunities for the development of industrial microwave foaming processes are identified, and a roadmap for potential future research works is defined.

## 2. Principle, Mechanism, and Steps in the Microwave Foaming

**Figure 1:**
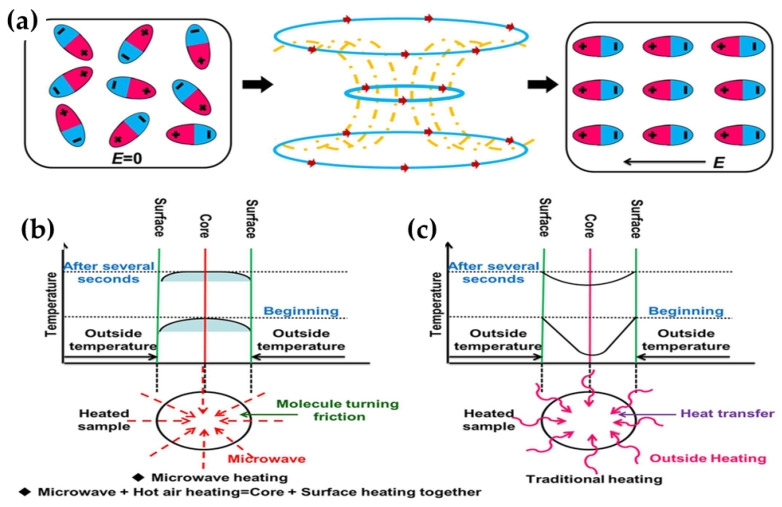
Mechanism of microwave heating (**a**,**b**), and traditional heating (**c**). Reprinted with permission from the authors of [13]. Copyright © (2015) Nature research.

In the last two decades, microwave foaming processes have been increasingly adopted for the production of starch-based foams [21], phenolic foams [11], EPS, and EPS-syntactic foams [23,63], and metallic foams [21,24]. This section will highlight the foaming steps and the mechanisms for producing foams using microwave heating. Table 2 summarizes the different microwave foaming processes with its physical, mechanical, and thermal characteristics of the resultant foamed products. The main advantage of microwave heating in foaming applications is that it heats the material volumetric, which is critical during expansion. Heat is generated by the interaction of polar molecules with the alternating microwave electromagnetic field. According to Fenghua Zhang and co-workers [13], this interaction continuously induces frictional loss due to polar molecules, converting electromagnetic energy into heat (Figure 1a). Therefore, compared to conventional heating, which is limited by heat transfer through the material’s surface, microwaves can penetrate the materials causing uniform temperature distribution within the entire material, subject to penetration depth (Figure 1b).

In general, the microwave foaming processes involves three main steps: (i) Mixing of material with blowing agents. Water has been principally used as a blowing agent to produce polymeric foams, thermoset, and starch-based foams. This is because of its good microwave absorbing capacity. On the other hand, other blowing agents like sodium bicarbonate (SB), 4, 4-Oxybis (benzene sulfonyl-hydrazide (BSH), and azodicarbonamide (ADC), are also used for microwave foaming reaction. (ii) Nucleation or expansion of material during microwave irradiation. This is an important step that directly affects the cellular foam structure. The expansion of foam during microwave processing can be controlled by optimizing the microwave processing parameters, which are comprehensively discussed in Section 3. (iii) The stabilization of foam’s cellular structure using drying or curing. The detailed description of microwave foaming steps of different materials is described in the following sections. 

### 2.1. Starch-Based Foams

**Figure 2:**
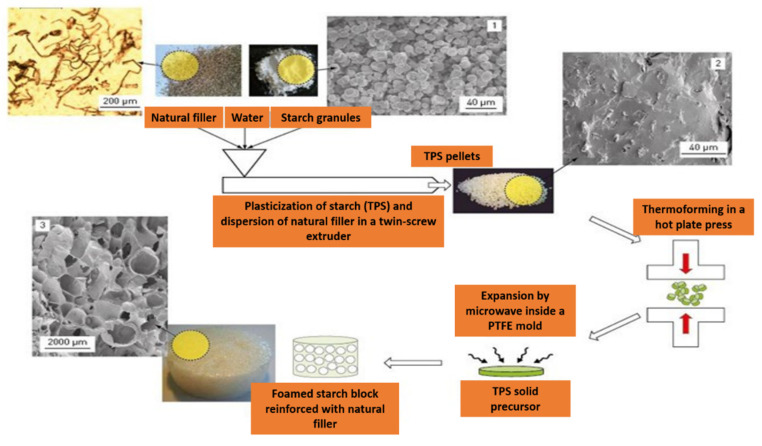
Microwave foaming process of starch foam from extruded plasticized starch [77].

Starch-based foams are produced by microwave processing by using either plasticized starch (processed through extrusion) [9,15] or starch-containing water batter (which eliminates the production step of extrusion) [27]. The microwave expansion can be performed in either a PTFE cavity to create a molded part or free expansion. Figure 2 shows the fabrication steps of starch foam via microwave foaming of extruded plasticized starch as reported by Moraru and Kokini [70]; the actual microwave foaming mechanism of starchy matrix progress is as follows. Initially, the applied microwave energy selectively heats the water, increasing the temperature of the matrix volumetrically. As the temperature rapidly increases, the water generates homogenous superheated steam, which serves as a driving force that creates high pressure inside the starchy matrix. Afterward, the matrix starts to expand (nucleate) under superheated pressure due to its transition from a glassy state to a rubbery state. The nucleation and bubble growth during microwave foaming are the key steps to attain the required characteristics of foams. Their behavior depends on the factors discussed in the section below. According to Moraru and Kokini [76] and Shafi et al. [74], bubble growth is directly related to the foams’ pore structure and pore distribution. The formation of vapor bubbles is derived by superheated steam pressure inside the matrix, which is controlled by surface temperature and moisture content of the matrix [45]. S. Kraus et al. [64] reported that increasing microwave power leads to faster removal of water during the foaming process that, in turn, increases the number of vapor bubbles and decreases the pore size of the product. This finally results in more expansion of matrix compared to low microwave powers. In addition, during bubble growth, significant water loss occurs due to the escape of water vapor through the bursting or thinning of cell walls. This transforms the polymer matrix into a glassy state, stabilizing the cell structure [46].

### 2.2. Polystyrene and Syntactic Foams

**Figure 3:**
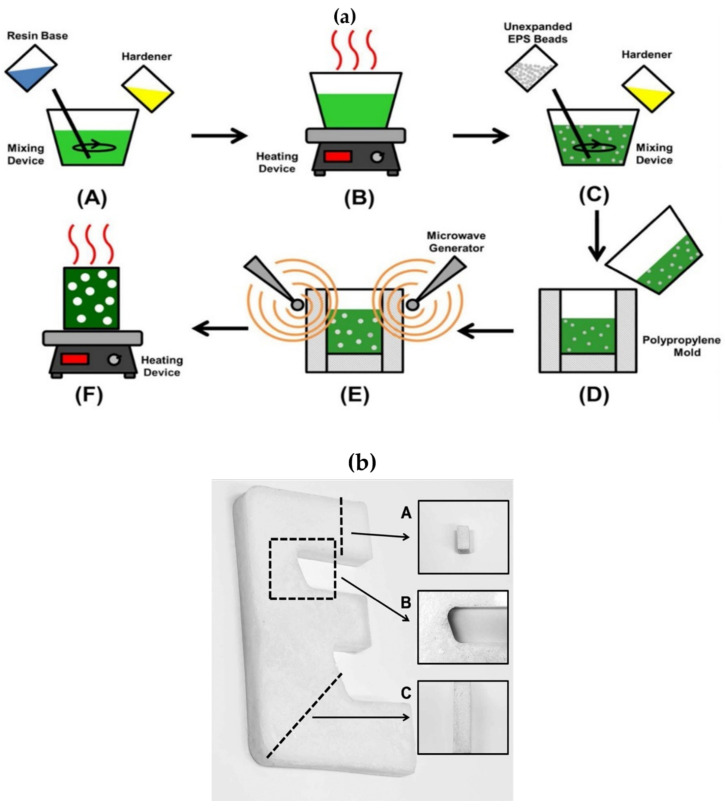
(**a**) Experimental setup for microwave expanding of epoxy-EPS syntactic foam: (A) mixing epoxy base and the first-part hardener, (B) pre-curing, (C) mixing unexpanded EPS microspheres, the second-part hardener, and the procured epoxy, (D) transferring foaming feed to a polypropylene mold, (E) microwave heating, and (F) post-curing]; (**b**) EPS–epoxy syntactic foamed letter “E” part molded via microwave expanding process: (A) cross-section of the upper arm, (B) magnified image of the upper curving section, and (C) cross-section of lower curving section. Reprinted with permission from the authors of [70]. Copyright © (2015) Society of Plastic Engineers.

The highly efficient and fast manufacturing of polystyrene foams revealed microwaves as a promising potential technique in the polystyrene industry. Calles-Arriaga et al. [43] formed polystyrene foams using microwave heating of EPS microspheres with three different solvents (ethanol, ethanol/water, and hydrogen peroxide). Similarly, the production of syntactic foams with EPS microspheres was also studied using microwaves [44,70,78]. Usually, solvents with high dielectric loss (such as water) are expected to exhibit better foaming under microwave heating. Three techniques generally produce EPS microspheres-syntactic foams; blending EPS with resin, expanding EPS during the curing process, and expanding EPS using thermal energy. However, all these techniques are limited due to the low extent of foaming and non-uniformity of the foamed structure [79,80]. In 2015, Yifeng Hong et al. [70] proposed microwaves as favorable for processing syntactic foams with high EPS loading. This is due to microwaves’ advantages to penetrate the bulk of the material, overcoming heat transfer limitations, and more even foaming derived from volumetric heating. Moreover, a special feature of microwaves in this application is auto-limiting heating, which means that the material becomes less microwave absorbent once a foaming reaction occurs, and thereby, microwave energy will be preferentially absorbed by unreacted parts of the material [81]. Figure 3a shows the schematic diagram of EPS/epoxy syntactic foam fabrication using microwave heating. The author [70] studied the microwave foaming process’s feasibility and optimized three parameters (microwave power, epoxy viscosity, and heating time). As shown in Figure 3a, the first step involved mixing a resin base and curing to achieve the desired mixture viscosity, which is the key parameter to produce the required surface structure of the product, as discussed in detail later. In the second step, EPS beads and hardener were mixed with resin. The purpose of hardener is to prevent dissolution and facilitate the homogenous distribution of beads. The third step is foaming of the mixture inside the mold using microwave heating and curing to stabilize the structure. The processing of foaming and curing is completed in few minutes, suggesting a fast and potentially energy saving process. During foaming, the foams can be easily molded into complex shapes such as “E” when microwave heating is employed (Figure 3b). The pressure generated during microwave foaming act as a driving force to achieve an efficient molding process successfully, ensuring sufficient adhesion or bonding between spheres. The resulting foam product exhibited smooth surface and homogenous distribution of pores, confirming an effective foaming process during microwave heating.

**Figure 4:**
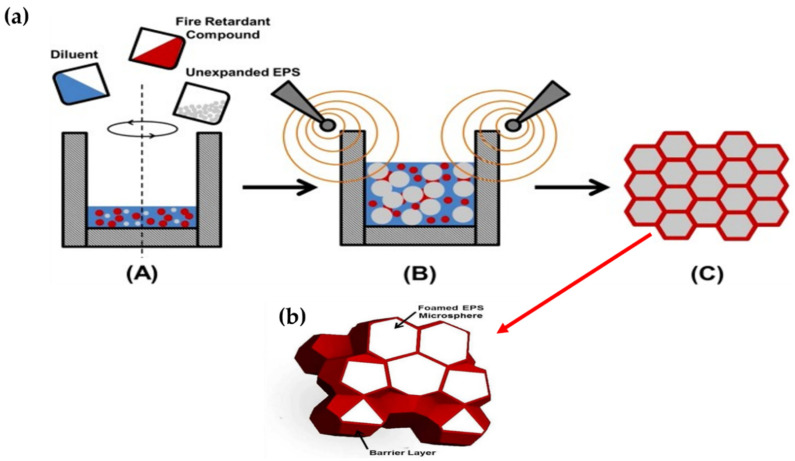
(**a**) Process setup and procedure to produce composite EPS foam from an expandable suspension; (**b**) composite EPS foam, a 3D pictorial model with cross-section. Reprinted with permission from the authors of [44]. Copyright © (2015) Society of Plastic Engineers.

Similarly, Yifeng et al. [44] reported the fabrication of honeycomb-like EPS structures filled with fire retardant syntactic foam using microwave heating, as shown in Figure 4a. The process includes homogenous mixing of diluent, fire retardant, and unexpanded EPS followed by microwave heating, resulting in maximum expansion at 1000 watt 2.45 MHz and 3 min. Figure 4b shows a thin barrier layer, which is due to the high loading of EPS during microwave foaming. This considerably enhanced the fire retardancy of the product without affecting the other foaming properties.

### 2.3. Phenolic Foams

**Figure 5:**
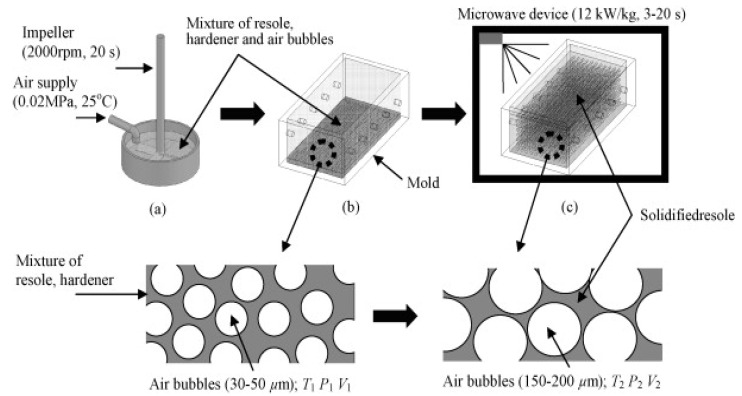
(**a**). Microwave foaming method: (**a**) mixing resole and hardener with air bubbles embedded, (**b**) injection to mold, and (**c**) microwave foaming. Reprinted with permission from the authors of [15]. Copyright © (2008) Elsevier.

Phenolic foams produced via microwave processing effectively overcome several environmental concerns linked to conventional foaming processes. During extruded foam production, phenolic foams typically require chlorofluorocarbon or hydro-chlorocarbon as a blowing agent. These substances pose a serious threat to the environment due to their high global warming potential. In addition, during foaming, these phenolic foams generate water as a byproduct, and this requires a simultaneous curing process, which is challenging to achieve uniformly using traditional heating. In 2008, Kim et al. [15] proposed and verified the fabrication of high-density phenolic foam (approx. 100 kg/m^3^) using microwave heating. The presence of polar molecules in the phenolic resin can effectively absorb microwave energy, which led to a uniform and quick increase in temperature of the resin and resulted in the uniform cellular foamed product. The favorable process step is that, as the temperature goes above 100 °C, the byproduct water and alcohol solvent start to escape from the foam matrix. This allows air to embed in the foam pores and thus significantly reduces the thermal conductivity of foam. Figure 5 shows the schematic diagram of the foaming process of phenolic foams. The process involves three main steps: (i) air bubbles embedding with resoling and hardener using an impeller, (ii) injection in the mold, and (iii) microwave expansion [15]. The idea of an embedded air bubble is due to the high viscosity of resoling, which prevents maximum expansion during foaming. As shown in Figure 5, the resole is expanded about 5–10 times in the microwave foaming process to achieve low-density foam. This expansion is linked to the resole’s temperature, which needs to reach 150–250 °C to produce a cellular product. Moreover, the presence of large voids is associated with a substantial increase in water volume to about 1000 times when it changes from liquid to gaseous phase during microwave heating at high temperatures. The author also attributed this phenomenon to the presence of unreacted phenols in the foam matrix.

A similar methodology was adopted by J. Choe et al. [47] to fabricate low-density phenolic foam using microwaves in the presence of chopped glass as a reinforcing material. A closed mold foaming was employed, and the average uniform foam density reported was 35 kg/m^3^ and thermal conductivity of 0.039 Wm-K, which were within a suitable range for insulating applications. The presence of chopped glass significantly enhanced the mechanical properties of foam without altering the insulating performance. Song et al. [68] studied the incorporation of carbon nanoparticles on phenolic foam using microwave heating from which they investigated the effect of carbon particles on the formation of the pores during foaming by changing resin viscosity and cross-linking.

### 2.4. Modified Microwave Foaming Processes

**Figure 6:**
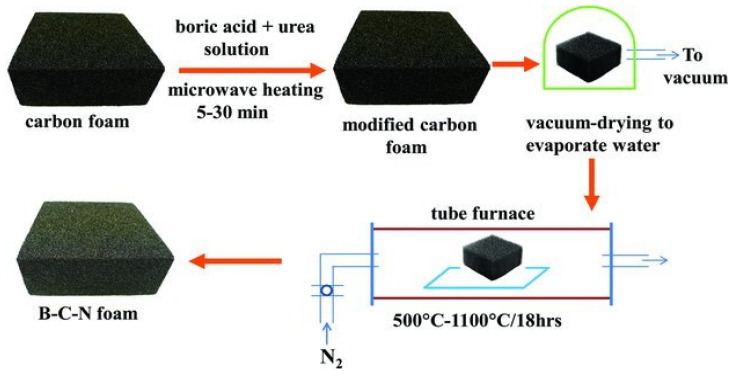
Schematic synthesis procedure of B-C-N foam from carbon foam. Reprinted with permission from the authors of [74]. Copyright © (2012) John Wiley & Sons.

These techniques have been recently applied to produce different types of highly porous and adsorbent foams, such as B–C–N and NiCr foams. In this regard, Rajib Paul et al. [74] utilized a combined microwave–chemical processing technique for the surface treatment of carbon foams to produce boron-carbon-nitrogen (B–C–N) foam. This method involves the successful conversion of carbon foams to boron carbon nitride (B–C–N) by microwave heating carbon foam in the presence of boric acid and urea solution, as depicted in Figure 6. Microwave influence on effective initiation of surface chemical reaction and annealing at 900 °C temperature resulted in the formation of foams containing high porosity and right stoichiometry between BC_2_N and BC_4_N. The B–C–N is a hexagonal porous solid exhibiting a large surface area and high thermal conductivity, which is a suitable substrate for thermal energy storage and adsorption of hydrocarbons.

Microwave hybrid heating was adopted by Kangjian Wu et al. [45] to process NiCr foam by pyrolysis of polyurethane. These types of metallic foams are commonly produced by pyrolysis of polyurethane foams using conventional heating [82,83]. However, compared with conventional heating, using microwave-hybrid technology significantly reduces the processing time and residual carbon content in pyrolyzed foams—however, the formation of burst holes is due to the rapid degradation of polyurethane during microwave irradiation. The purpose of microwave hybrid heating is to overcome the homogeneity challenges associated with microwave systems. Usually, indirect microwave heating of metallic foams consists of sharp edges; the microwave energy concentrates at the foaming material [84], leading to the ignition of gas plasma (arcing) and overheating and melting of the material. Therefore, a combination of direct microwave heating with external coupled heating containing microwave susceptors such as SiC (high dielectric loss at low temperature) in the foaming process. The SiC susceptor weakens the microwave intensity at the sharp edge by efficiently absorbing microwaves, and this reduces the gas plasma ignition during microwave foaming.

## 3. Effect of Microwave Foaming Influential Factors on the Final Properties of Foamed Materials

**Figure 7:**
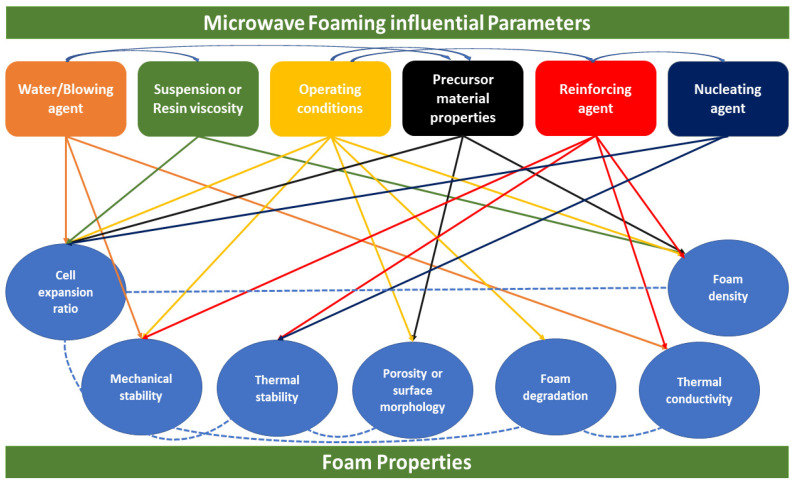
Microwave foaming influential parameters are affecting foam product properties.

The final characteristics of microwave foamed materials such as density, thermal conductivity, expansion ratio, cellular structure (i.e., cell size, pore distribution, and porosity), and mechanical and thermal strength are greatly influenced by various microwaves operational factors. The key factors include water/moisture content (or blowing agent of foaming material), the viscosity of foaming suspension, microwave power (or power density), and time. In addition, precursor characteristics, including dielectric properties, blend ratios, and incorporation of additives, are also important. Therefore, this section comprehensively discusses the impact of these parameters on the microwave foaming process and their influence on the final microwave foamed materials’ physical and thermal properties. A schematic illustration representing the microwave parameters effect on final foam properties is shown in Figure 7. This is expected to enhance readers’ understanding of the microwave foaming mechanism and help comprehend the effect of altering or controlling these key influential factors on produced foamed characteristics.

### 3.1. Effect of Water or Blowing Agent Content

**Figure 8:**
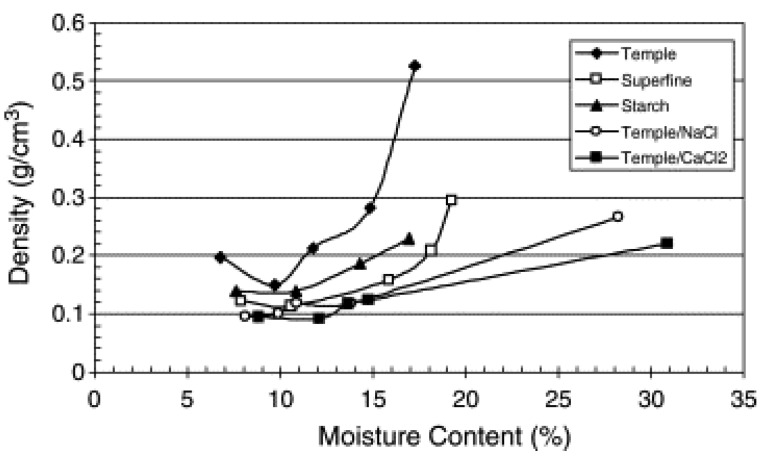
The densities of pellets microwave-foamed at different moisture contents. Reprinted with permission from the authors of [48]. Copyright © (2006) Elsevier.

In microwave foaming processes, water content is an essential process parameter and is commonly employed as a blowing agent or plasticizer for the production of different foaming materials. During the microwave foaming process, water molecules are the main source of dipole movement and therefore provide a rapid increase in temperature due to its molecules’ vibration. It is thought to produce superheated steam, which is the main driving force to induce the expansion of foaming materials. According to previous studies [15,51,59,85], the percentage of water content before foaming or water loss during foaming directly affects the number of properties of the foamed product, including expansion ratio, density, and cellular structure, thermal conductivity, and mechanical properties. Many researchers have assessed the effect of water content or water loss (weight loss) before and during microwave foaming on microwave foams behaviors. According to the study credited to [86] Sjöqvist et al., the initial achieved foam expansion was low and was insufficient at low water content. However, a further slight increase in water content significantly enhanced the foam’s expansion, producing porous and small density products. It was demonstrated that the water content (RH) > 60%) resulted in a continuous decrease in the expansion, which the author attributed to the collapse of the formed foam cell after expansion. Similarly, as seen in Figure 8, the lowest density foams are obtained at 10% *w*/*w* moisture content for all the samples. An increase in the foam density by >10% may be attributed to the reduction in expansion ratio due to loss in water during heating, a lower degree of selective heating of the water due to increased water content (i.e., lower power density in the water phase leading to lower steam pressure), and formation of a denser skin layer.

**Figure 9:**
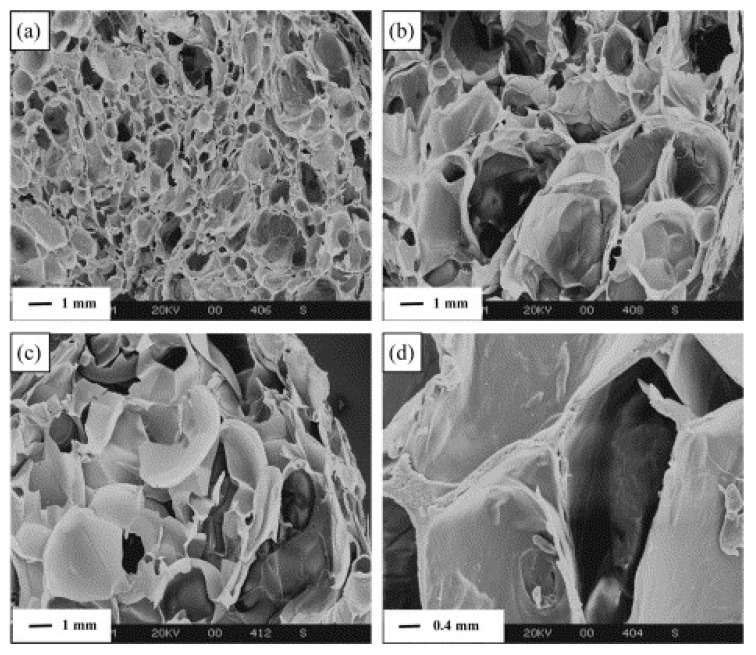
(**a**) Scanning electron micrographs of foamed temple pellets with various additives: (**a**) Temple; (**b**) Temple/BIH (1.5% *w*/*w*); (**c**) Temple/talc (0.8% *w*/*w*); (**d**) Temple/talc (2.2% *w*/*w*). Reprinted with permission from [48]. Copyright © (2006) Elsevier.

This behavior is also confirmed by the micrograph analysis of low- and high-water content foamed products. Sjöqvist et al. [86] reported that the highest porosity achieved at 33% RH; however, high moisture content (75% RH) resulted in a collapsed cell and thick skin. Similarly, 10% *w*/*w* moisture content showed the lowest density of 0.114 g/cm^3,^ which was increased with a high moisture content of foaming material (Figure 8). The author attributed this observation to the plasticizing effect, which reduces the tension generated inside the cell’s walls during foaming. This possibly led to the reduction in expansion and also eventual collapsing of some cells, as confirmed by a previous study [86]. As proved by Jia Zhou et al. [48], the larger the water vapor pressure, the more the expansion and bubble growth burst and achieve lower density and improvement in fraction of open cells, which can be controlled by process parameters and additives. An additional blowing agent could be utilized further to enhance water vapor pressure [48]. Figure 9 illustrates the SEM micrograph of temple pellets with Hydrocerol^®^ BIH and talc as additive. The micrographs result indicates no difference with or without BIH blowing agent because of the insufficient decomposition of the blowing agent during microwave foaming (Figure 9b). However, the incorporation of talc powder has substantially reduced the size of the foam cells. The cell section diameters are in the range of 0.2–0.8 mm and 50 μm to 0.5 mm for 0.8% *w*/*w* talc powder and 0.2% *w*/*w* talc powder foam, respectively.

The percentage of water loss during microwave foaming was also investigated in some previous studies, which indicated a significant influence on the final foam product’s characteristics. Usually, during microwave foaming, increasing the material’s temperature leads to the generation of steam inside the matrix. Most of the steam escapes from the foam cell structure rather than being retained inside the cells. This leads to expansion and loss of weight (or loss of water) of material, which develops changes in the produced foam’s properties. For microwave foaming of starch-based materials, water, acting as the blowing agent, also acts as a plasticizer.

Additionally, the continuous escaping of water from the matrix leads to expansion and a simultaneous increase in the final foam product’s stiffness to stabilize the cellular structure that forms during microwave processing. Due to water loss, there is an increase in the glass transition temperature, and when it reaches greater than foam temperature, maximum foam expansion is achieved, and the structure of the foam is then stabilized [87].

Moreover, the water loss during microwave foaming results in a considerable increase in the foamed product’s mechanical properties compared to the precursor. Using the cubic cell model proposed by Gibson and Asley [88], the estimated compressive modulus of starch foam by A.Lopez-Gil et al. [51] is found to be 50 times higher than the solid precursor. Flor Concenia and coworkers [56] reported that the presence of water in the foaming system of inorganic materials contributed well to homogenization and adsorbed within the micropores of materials, which helps in the formation of the macro-porous surface of foam because of its slow release during microwave foaming.

Bu Gi Kim and Dai Gil Lee [15] successfully fabricated low density and conductivity phenolic foams using air bubbles as blowing agents via microwave foaming techniques. The results (Figure 5) showed both open and closed cells at the center and closed near the ends. The authors associated large voids in foam structure due to water content evaporation from resoling during microwave foaming. Due to an increase in temperature, there is a substantial increase of about 1000 times in water volume when it changes from liquid to gaseous phase. The significant increase in volume during microwave foaming results in a drastic reduction of density and thermal conductivity of phenolic foam as compared to other produced phenolic products. Similar results were also reported in other studies [47,89]. Other workers also utilized other blowing agents such as pluronic, pentane, dichloromethane, sodium silicate, etc. during microwave foaming of different foamed materials (Table 2). Calles-Arriaga et al. [43] performed microwave foaming of polystyrene using ethanol, ethanol/water, and hydrogen peroxide as blowing agents. The authors revealed that, during EPS foaming, ethanol or ethanol/water promotes a sharp increase in temperature. However, the process produces moisture content as a byproduct, which requires extra microwave heating and the resultant foams exhibited easy detachment of EPS pearls. In contrast, using hydrogen peroxide instead facilitates low water content and strong attachment of the EPS pearls.

### 3.2. Effect of Suspension/Resin Viscosity

Viscosity is another factor with a significant impact on the final properties of materials foamed via microwaves. Therefore, in order to improve the properties during microwave foaming, it is highly recommended to select suitable viscosity for the foaming suspension/resin. However, very few studies have been conducted to investigate the behavior of changing mixture viscosity during microwave foaming on the foamed product’s properties. Song et al. [68] reported that the resin viscosity for phenolic foams directly affects the cell density, indicating that the cell expansion can be controlled by resin viscosity. The authors further demonstrated that during microwave foaming, as the cross-linking begins, there is a strong enhancement in the resin viscosity, which hinders the pores’ expansion. It was demonstrated that the incorporation of nanomaterials such as MWCNT caused a significant increase in resin viscosity, which showed large cells, presence of unblown particles, and high density foamed products [27]. However, graphene presence in reinforced phenolic foams showed much lower density and improved compressive strength, which was attributed to better and uniform graphene dispersion and lower resin viscosity [68].

**Figure 10:**
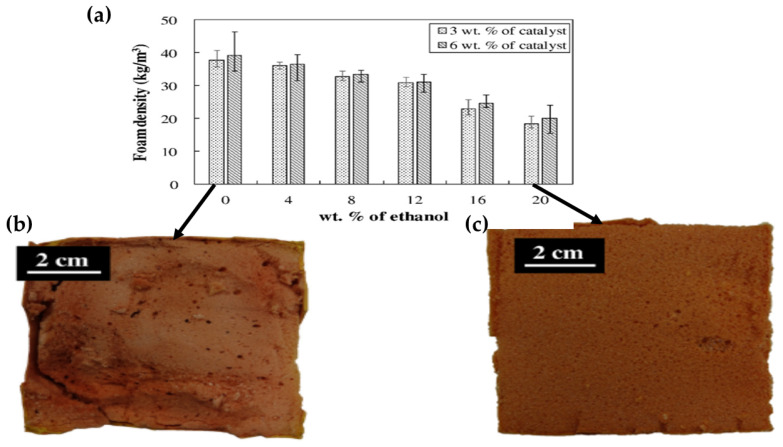
(**a**) Free rise phenolic foam density concerning the wt.% of ethanol; (**b**,**c**) photographs of the phenolic foams’ cross-sectional views with three wt.% of PTSA catalyst concerning the wt.% of ethanol, 0 wt%, and 20 wt%. Reprinted with permission from the authors of [47]. Copyright © (2016) Elsevier.

Using ethanol to control the resin viscosity, Jacheon et al. [47] studied the effect of resin viscosity on the phenolic foam’s density and cell structure and the percent of ethanol on the foam density. The results displayed in Figure 10a indicate that the foam density decreased from 37.7 kg/cm^3^ to 18.3 kg/cm^3^ when 20 percent ethanol was added. The author associated this variation with a significant reduction in resin viscosity at higher ethanol content, which considerably enhanced pores’ expansion. According to the Rayleigh–Plesset equation [90], the velocity of bubbles formed during the microwave expansion process is inversely proportional to resin viscosity. Therefore, based on the hypothesis, for a low viscous resin, the expansion is more significant in a shorter time, producing larger cell size and lower foam density. Moreover, due to the broader expansion of cells, the low viscous resin foam’s surface exhibited rough morphology compared to the smoother surface of high viscous resins as illustrated in Figure 10b,c, respectively.

### 3.3. Effect of Microwave Operating Variables

Microwave operating parameters such as microwave power, exposure/heating time, and modified microwave foaming techniques—such as chemical assisted microwave or hybrid microwave heating—also have considerable influence on foamed products’ density and cellular structure, which directly reflect the mechanical and thermal characteristics of foams. In 2012, Alexander et al. [69] studied the change in microwave heating time on the density, surface morphology, and strength of PU foam products. The authors reported that the increase in microwave exposure time at fixed power significantly altered their produced foams’ strength and elasticity. Similarly, in 2013, S. Kraus et al. [66] investigated the effect of microwave power and system pressure on the expansion during the foaming process, with another study corroborating their findings [87]. It is indicated that the volume expansion increased with an increase in microwave power attributed to the linear increase in temperature inside the foaming material with an increase in microwave power, as supported by other previous studies [91,92]. Due to the high microwave power, the author concluded that the temperature inside the material was higher, which led to high vapor pressure and, hence, the enhanced volume expansion. Xi Peng et al. [64], evaluated the change in a dielectric loss concerning microwave power. It was observed that at a higher microwave power range of 150 W/g–200 W/g, dielectric loss factor significantly jumped to over 0.3 even at a lower system temperature of 25 °C and remained at a steady-state up to temperature 160 °C. However, at a short microwave power range of 75 W/g to 150 W/g, the dielectric loss remained significantly low between 0.05 to 0.1 and increased when the process temperature was greater than 80 °C. In another similar study [65], microwave power’s effect on the pore distribution was evaluated. The pore distribution results showed that there is a significant increase in the nucleation rate of vapor bubbles at high microwave power, which led to a significant increase in the number of pores within small areas. By employing FTIR characterization, Demitri et al. [71] investigated the influence of microwave power and time using a highly interconnected and homogenous structure CS-PEGDA scaffold. It was revealed that the higher microwave heating time, i.e., 240 s, possibly started the scaffold’s thermal degradation as confirmed by the yellowing color of the product. Moreover, the microwave curing power was found to be independent of the reaction yield. In another study, the surface roughness of the final foam product was increased with increasing time of microwave chemical assisted foaming of carbon foams [74]. It is suggested that the increase in surface roughness can be attributed to the breakage of carbon bonds and creation of defects at more exposure time.

### 3.4. Effect of Foam Precursor Materials Properties

**Figure 11:**
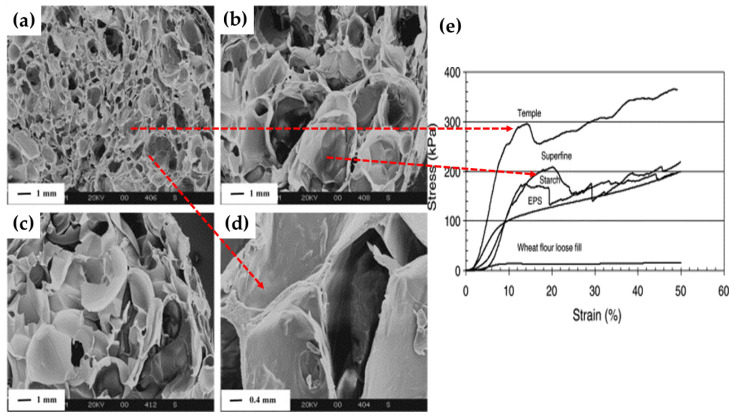
Scanning electron micrographs of cross-sections of foamed pellets with different raw materials: (**a**) temple flour, (**b**) superfine flour, (**c**) purified wheat starch, and (**d**) cell wall in temple foam shown in (**a**) at higher magnification. (**e**) Comparison of typical compressive stress–strain curves (22 °C, 50% RH) for the microwave-foamed pellets made from different raw materials with commercial protective packaging materials. Reprinted with permission from the authors of [48]. Copyright © (2016) Elsevier.

Foam precursor materials such as composition, molecular weight, dielectric properties, melt viscosity, and blend ratios in composite foams reflected considerable influence on the final physical properties such as density, cell wall thickness, and cell pore size, and mechanical and thermal stability of the foam. For instance, Jiang Zhou et al. [48] investigated three precursors (temple, superfine, and wheat starch) for producing starch-based foams via microwave. Based on morphology results, the temple starch-based foam material exhibited adequate cell structure associated with the presence of bran (outer layer of grains) in temple starch, which acts as a natural nucleating agent during the microwave foaming process. Other studies also reported similar behavior when foamed material using different techniques [93]. Apart from the composition of precursor materials, another essential characteristic is the foaming material’s dielectric properties. Understanding change in dielectric constant (ε’) and loss (ε″) is critical to understanding the fundamental mechanistic interactions between microwave energy and materials. Generally, during microwave heating, the increase in temperature inside the materials is due to the dielectric polarization of material indicated by its dielectric loss factor [93,94]. The dielectric loss factor (ε″) is defined as the efficiency of material to convert the electric energy to thermal energy. A high dielectric constant means high microwave energy absorption capability [62]. The dielectric loss (ε″) in the range of 0.05 to 5 of a material indicates that the material can be foamed using microwave energy [46]. In several studies, the authors of the respective studies have reported the dielectric properties of foaming material to understand the microwave foaming mechanism. Peng et al. [64] investigated the dielectric loss of dry starch and extruded starch material (at different moisture content) at an increased temperature rate of 15 °C/min using controlled microwave foaming. The author reported no change in the dielectric loss factor of dry starch. The sharp jump of ε″ at temperature defined as T_ε_ of extruded starches is observed at the temperature corresponding to close to glass transition temperature (T_g_). Therefore, the temperature 100–110 °C above T_ε_ is the actual foaming temperature of extruded starches. As the temperature reached above T_g_, the mobilization of amorphous starch segments started and promoted easy dipoles to orient along the electric field’s direction. This resulted in more conversion of electric energy to thermal energy due to intermolecular friction during the oscillation of dipoles, which lead to the quick rise in ε″. In another study, the dielectric properties of a starch material were investigated at different moisture contents and were correlated with the porosity of foamed product [66]. The study reported that the dielectric properties showed increasing trend with an increase in moisture content which resulted in high expansion in foam cell volume and a more porous product. Moreover, as seen in Figure 11a–d, Jiang Zhou et al. 48], observed different cell structure and size of foam produced from Temple flour, superfine flour, and purified wheat starch. Higher proportions of large cell sizes (0.5–4 mm) are found in superfine and purified wheat flour. However, temple flour exhibited finer and smaller cell size (0.2–2 mm) foam structure associated with the presence of bran, which acts as a nucleating agent. It is interesting to note that temple starch’s compressive stress is higher than that of superfine and wheat flour foams. Commercial foams such as EPS (as a cushion block) and a wheat flour loose-fill used for protective packaging exhibited lower compressive strength than that of temple starch foam produced via microwave heating (Figure 11e).

### 3.5. Effect of Reinforcing Agents

**Figure 12:**
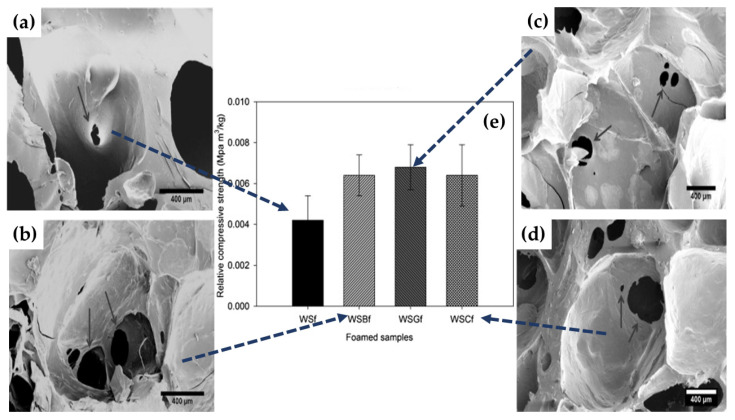
SEM micrographs of cells interconnected. (**a**) Wheat starch (WSf) (60×), wheat starch starch—grape—barley (WSBf) (60×) (**b**), (**c**) wheat starch—grape (WSGf) (40×), and (**d**) wheat starch—cardoon (WSCf) (40×). (**e**) Relative compressive strength of the foams produced. Reprinted with permission from the authors of [51]. Copyright © (2015) Elsevier.

The foamed product’s mechanical and thermal stability is another crucial property in its application in various fields such as packaging, construction, automotive, etc. [95,96,97]. Therefore, it is highly essential to fabricate foams with reduced density and better stability in strong and rigid structures. To achieve this, various reinforcing agents, which include natural fibers, carbon materials, and other nanoparticles, have been incorporated into the foaming material to enhance product stability and understand their effects on the foaming process and properties of foam products. The selection of a proper reinforcing agent is also crucial to achieving better interaction and homogenous dispersion within the foaming matrix to produce effective reinforcement without altering the other foamed products’ other properties. Lopez et al. [51] reinforced starch foam using three different types of natural fibers (barley, grape, and cardoon) (Figure 12). Effective embedding of all three fibers with the starch during microwave foaming resulted in foam with improved strength and rigidity comparable to that of pure starch foam. As seen in Figure 12a–d, the expansion of foam and cell size was lowered due to fillers’ addition. Several cracks and holes within the cell walls for all the produced foams implying the strong interconnected cellular structure of the foam. This is attributed to the heterogeneous nucleation rate/better interaction with starch matric that induces smaller cell size [76] and thus produced a more robust foam structure than others.

Carbon nanomaterials exhibit high mechanical and thermal strength and attracted greater attention to use as a reinforcing agent in different foaming materials [98,99]. This carbon material class also exhibited a high dielectric loss factor and can be a potential candidate as a nucleating agent during microwave foaming [100]. Song et al. [52] improved the toughness, strength, and thermal conductivity of phenolic foam using AC. Studies have reported that AC’s presence within the phenolic resin didn’t only induce a reinforcing effect but also acted as a barrier for escaping water molecules during the microwave foaming process. As a consequence, it significantly facilitated an increase in the cell size and reduction in the density of the foam and more satisfactory cell walls. However, higher content of AC is susceptible to agglomeration in phenolic foams. It tends to lead to the degradation of foam products due to a large amount of heat generation upon exposure to microwave radiation [100,101]. In a similar study, carbon black was used along with a blowing agent to produce PU foam. The study found that carbon black presence promoted the formation of a uniform, fine cell structure in larger quantities compared to neat polyurethane foam. This can be ascribed to improved microwave absorption due to the incorporation of carbon black, leading to more effective heating and benefiting the finer cellular structure [69].

**Figure 13:**
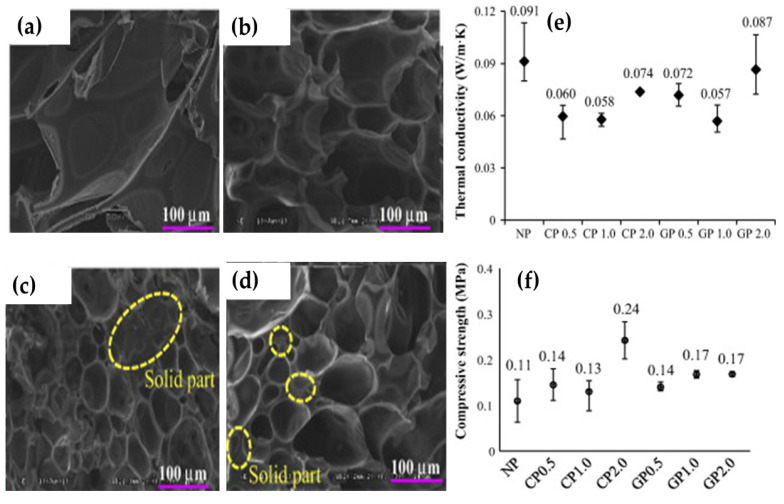
Scanning electron microscope images of (**a**) neat phenolic foam (NP), (**b**) phenolic foam with 0.5% multi-wall carbon nanotube (CP 0.5), (**c**) phenolic foam with 2% multi-wall carbon nanotube (CP 2.0), (**d**) phenolic foam with 2% graphene (GP2.0), (**e**) thermal conductivity, and (**f**) compressive strength of produced foams with respect to the particle type and weight fraction. Reprinted with permission from the authors of [68]. Copyright © (2014) Elsevier.

However, when MWCNT and graphene were utilized for improving the stability of phenolic foam produced by microwave energy, there was a decrease in the dissipation factor of phenolic foam containing MWCNT and graphene compared to neat phenolic foam. An increase in phenolic resin’s viscosity by incorporating nanoparticles can restrict the dipole movement and thus effected the cell formation and reduction in expansion during microwave foaming. The catalytic reaction of MWCNT with phenolic resin induced by microwave heating also causes cross-linking and thereby decreases the rate of gas expansion during microwave foaming and resulted in interconnected cellular structure (Figure 13b). The addition of MWCNT and graphene in appropriate amounts significantly improved the mechanical and thermal conductivity (Figure 13e,f). Moreover, Figure 13c,d showed aggregates within the polymer matrix. Air bubbles trapped between the nanoparticles expand dramatically and create a large bubble due to intense microwave absorption and lead to large cavities in the foam structure [68]. To overcome the viscosity problem during microwave foaming of phenolic foams containing reinforcing agents, Choe et al. [47] utilized ethanol to foam chopped glass fiber (3 mm)—phenolic foam. There was an approximate, 17 percent increase in tensile strength with 1 wt% chopped glass fiber without disturbing the physical and thermal properties of foamed product.

### 3.6. Effect of Additives/Nucleating Agents

Additives and nucleating agents improve microwave foaming behavior by absorbing microwave energy to provide effective heating within the foaming matrix. They also help to produce bubble growth by enhancing bubble pressure inside the matrix. Various types of additives such as salts, talc, iron oxide, glycerol, and PVOH have been utilized and also investigated to evaluate their effects on microwave foaming behavior and final properties of foam products. It is necessary to determine the appropriate mass of additives and its uniform distribution within the foaming matrix- and viscosity changes that lead to significant negative alteration in foaming behavior and finished foam properties. Improved microwave absorbing properties using additives allow increasing materials temperature, which causes decomposition of a blowing agent and leads to cellular foams’ formation. Therefore, non-homogenous distribution of additives/microwave absorbers or their agglomeration causes the degradation of foam, attributed to localized heating and eventual cell collapse [58]. Moreover, the addition of additive may alter the viscosity and the glass transition temperature of foaming materials. These changes in precursor properties lead to a decrease in the movement of molecules during microwave foaming. As reported by Peng el at [43], they require high energy for expansion while using glycerol and PVOH for the production of starch foams.

## 4. Discussion: Towards the Development of Industrial-Scale Microwave Foaming Processes

A comprehensive explanation of the theory of microwave heating and its application to materials synthesis are well-defined and are available elsewhere in the literature [58]. The key parameters for foaming microwave processing and applications are dielectric properties (i.e., nature and extent to which materials interact with the microwave electric field at the molecular level); power density, the dissipation of the electric field within a material; penetration depth of the electric field into the material; and the overall electric field distribution. There are clear benefits and opportunities for microwave heating in foaming. There is currently a lack of understanding of these parameters, with little or no studies considering them in designing and developing experiments and microwave heating systems—for example, in the sample size of materials, the power and time variables, and the overall dielectric loading with microwave heating cavities.

As this review demonstrates, the application of microwave heating for the lab-scale production of foamed materials requires a multidisciplinary understanding of several fields, including polymer chemistry, materials science, chemical engineering, and manufacturing. Scaling up of microwave processes from the lab to industrial-scale needs understands these areas and dielectric characterization to inform microwave systems design. Due to the high cost of microwave technology compared to other processes, it is also critical to understand the value proposition for microwaves, i.e., what microwaves can do that no other technology can. Table 3 gives some example value propositions for microwave technologies that could apply to microwave foaming.

## 5. Conclusions and Future Recommendations

There have been significant advances in the application of microwave processing techniques to produce a variety of foamed materials over the last two decades. This has reduced process time, energy consumption, and solvent-free processing at the laboratory and pilot scale, avoiding environmental concerns while sometimes allowing significant improvements in product quality. The critical challenges in microwave foaming of materials indicate the future focus for research activities in this field to enable scale-up and adoption within manufacturing environments:

- Utilization of new or sustainable higher dielectric loss blowing agents, e.g., water or their new combinations, to achieve better dispersion and foaming of different matrix materials that are incredibly transparent to microwaves. This is expected to provide effective heating and bubble growth and resulted in low-density cellular foams while overcoming the reliance on less sustainable blowing agents such as HCFCs and pentane.
- Dielectric characterization of foaming materials, additives, and blowing agents is highly desirable to understand better the microwave interaction with these materials, the importance of foaming temperature, and the overall foaming process. This will underpin not only the formulation of matrix-blowing agent-filler systems but also the design of bespoke microwave heating applicators and scale-up to industrial production.
- Rheological measurements such as viscosity, melt strength, and elongation strength are also vital issues required for providing an in-depth understanding of ways for controlling foaming, bubble stabilization, collapse, and expansion ratios for the production of foams with the desired requirements. Coupling this understanding with dielectric characterization also provides an opportunity to refine microwave heating protocols further.
- Use of nanofillers/modified fillers for both reinforcement and as nucleating agents for facilitating the foaming process in stabilizing bubbles growth. This is expected to result in higher expansion, low density, and thermally and stable mechanical foams.
- Modified microwave foaming techniques such as chemical-assisted microwave foaming in helping to induce cross-linking, branching, or degradation before foaming to achieve better foaming reduces the risk of cell collapse, improved expansion, and lower density cellular morphology of product, which has thus far proved challenging in some microwave foaming applications
- Development of new composite structures, e.g., syntactic polymer composites which cannot be readily produced using conventional foaming technologies

## Figures and Tables

**Table 1:** A brief summary of methods used for the foaming of materials.

Method	Steps	Application	Advantages	Disadvantages	References
Extrusion	Mixing of blowing agent in a polymer matrix Nucleation at high temperature Cell growth Cell stabilization	Petroleum polymer foams (EPS, EPE, EPU), bio-foams (PLA, starch, cellulose).	Simple and continuous process High production volume Low cost Shorter times (2–15 min) Foaming process economically feasible at a larger scale Pre-molding is not needed Introduction of nucleating agents at any time	Poor control of porosity, non-uniform cellular structures Presence of residues in the final product High temperature and shear forces Long single or double (usually corotating) screws Required tools expensive depending on machine capacity	[9,18]
Extrusion with supercritical fluids	The solubility of the physical blowing agent (CO_2_, N_2_) in the polymer matrix at high pressure Creation of pores by vaporization of blowing agent. Stabilization of cell	Petroleum polymer foams (EPS, EPE, EPU), bio foams (PLA, starch, cellulose).	Poor control of porosity, non-uniform cellular structures. Presence of residues in the final product High temperature and shear forces Long single or double (usually corotating) screws Required tools expensive depending on machine capacity	High volatility of supercritical liquids Lack of understanding of the nucleation process High diffusivity Required single or double screws Required tools expensive depending on machine capacity	[19,32]
Freeze-drying/solvent exchange	Freezing Primary drying Secondary drying	Bio-foams (starch, cellulose) for tissue engineering scaffolds	The cellular structure is controlled by the the size and distribution of the ice crystals Microfoams at a high freezing rate (−196 °C) and macro foams at a low freezing rate (−15 °C) Moderate density forms 10^4^–10^11^ cells/cm^3^	Not applicable for all materials Low to moderate expansion	[19]
Microwave foaming	Mixing of blowing agent Microwave heating Cell growth Cell stabilization	Bio-foams Metallic foam Phenolic foam	Uniform heating Fine cellular structure and greater cell sizes No residues Reduction of solvents	Materials with good microwave absorption can be foamed High-density foam	[33,34]
Batch foaming	Saturating polymer with a blowing agent Separation of the mixture Cell nucleation Cell growth and stabilization Gradual foam structure formation	Thermoplastic polymer foams	Simple batch-wise process Cheaper than other methods Low material requirement Pre-molding to the desired form Sensitive materials processing as no shear Achievement of uniform cell size distribution Moderate to high expansion Screw not needed Medium to high blowing agent uptake	Limited to fundamental foam studies of new materials Fixation of foaming composition during pre-molding Higher density 106–1016 cells/cm^3^ compared to batch and extrusion methods 30 min to 72 h depending on polymer type and sample thickness Pre-molding is needed	[18,19]
Injection molding foaming	Saturating polymer with a blowing agent Separation of the mixture Cell nucleation Cell growth and stabilization Gradual foam structure formation	Thermoplastic polymer foams	Semi-continuous process Complex foam parts formation Lower density foam 104–108 cells/cm^3^ Cycle time shorter than the batch process The building of 1 to 100 microns uniform cell diameters Introduction of nucleating agents at any time	Pre-molding is not needed A medium to a large number of materials (in kilograms) is needed Supercritical fluid (SCF) as a physical blowing agent. CO_2_ and N_2_ are usually used as an agent Expensive tools depend on machine capacity Mean cell size determination is difficult Mechanisms of cell nucleation and growth not understood	[18,19]

**Table 2:** Summary of microwave foaming process and characteristics of foamed products.

Precursor	Blowing Agent	Additives	Operating Condition		Foaming Steps	Foam Product Properties				Remarks/Microwave Benefits	References
			Power (Watt)	Time (s)		Product	Density (g/cm^3^)	Porosity	Compressive Strength (MPa)/Thermal Conductivity (W/m K)		
Temple flour, superfine flour, purified wheat starch	Hydrocerol	Calcium chloride and sodium chloride, talc powder	-	50–65	Extrusion (12%–13% MC) Additive addition Microwave foaming	Temple Superfine Starch Temple/NaCl Temple/CaCl_2_ Temple/BIH Temple/talc (2.2%)	0.15 0.114 0.139 0.095 0.092 0.144 0.188		0.314/- 0.180/- 0.161/- 0.250/- 0.174/- 0.334/- 0.489/-	Effective microwave absorption due to the presence of salts led to the increase in heating rate, low moisture loss due to diffusion, and larger cell sizes. The mechanical properties of microwave foamed foam were found close to that of the EPS block.	[48]
Purified wheat starch wheat flour	Water	Glycerol, polyvinyl alcohol			Extrusion and addition of additives Microwave foaming	-	-	-		Glycerol enhanced microwave absorption efficiency and high heating rate at lower microwave power Presence of additives constraint to foam cell growth during microwave foaming	[64]
Purified wheat starch wheat flour	Water	Glycerol, polyvinyl alcohol	200			-	-	-		Reduction in expansion during microwave foaming of starch materials containing glycerol	[46]
Native corn starch	Water	None	400 600 800	120 120 120	Extrusion (MC 41%) Vacuum foaming and drying			2020 pores 1018 pores 1970 pores		Higher the microwave power leads to a higher number of vapor bubbles nucleated and showed increase in the volume expansion microwave Vacuum expansion allows an indirect expansion with lower time and energy consumption	[65]
Native corn starch	Water	None	400 600 800	120 120 120	Extrusion (MC 0.695 kg/kg) Vacuum foaming and drying		0.82 EI 0.97 EI 1.17 EI			The volume of starch-based pellets significantly increased with increasing microwave power; effective moisture diffusivities increased with an increasing microwave power.	[66]
Native wheat starch	Water	Barley straw fibers, cardoon waste, and grape waste. Barley straw fibers	900	50	Extrusion with and without additives Microwave foaming of sheets in PTFE mold	Starch Starch/Barley (95:5) Starch/Grape (95:5) Starch Cardoon (95:5)	0.292 0.347 0.301 0.303	cell sizes > 0.5	0.87/- 2.18/- 2.03/- 1.92/-	The microwave foaming process allowed the continuous production of foam blocks (without joining between pellets). This leads to about an 800-time increase in mechanical performance (stiffness and strength) compared to pellets microwave foaming.	[51]
Maize flour		Zein biopolymers	1000	15	Extrusion with and without additives (MC 26%) Microwave foaming	Starch Starch/zein/55/5 Starch/zein/85/15 Starch/zein/70/30	0.16 0.2 0.22 0.44	<100 um < 100 um < 100 um > 200 um		Foams from microwaved polymer mixture exhibited finer cellular structure compared to directly expanded material.	[67]
Carboxymethylcellulose	Pluronic	Polyethylene glycol diacrylate	900	105	Mixing of polymers and blowing agent Microwave foaming Drying					The microwave method effectively induced thermo-polymerization with time and energy savings. The foam is a hierarchical structure having open porosity of different sizes.	[57]
Resole	Air bubbles	-	12,000	3–20	Mixing of resoling, hardener, and air bubbles using an impeller Microwave foaming	Phenolic foam	0.12	100–150 um dia	-/0.029	Microwave significantly decreased the content of H_2_O (the byproduct of cure reaction), which leads to low conductivity foams compared to conventional phenolic foams.	[15]
Resole-type phenolic resins	Air bubbles	AC powder	12,000		Mixing the resole and accelerators with or without the AC using an impeller chemical cure reaction Microwave foaming	Phenolic foam Phenolic/AC (1 wt%)	0.127 0.103	233.8 um 169.6 um	1.68/0.064 2.17/−0.071	Microwave radiation helps chemical interaction between the phenolic resin and AC during foaming that induces a robust interface and thus resulted in a firmer foam.	[52]
Resole-type phenolic resins	Air bubbles	MWCNT and graphene	12,000	20	Mixing the resole and accelerators with or without the additives using an impeller chemical cure reaction Microwave foaming	Phenolic foam 0.5 wt% MWCNT 1.0 wt% MWCNT 0.5 wt% Graphen 1.0 wt% Graphene e	0.065 0.050 0.065 0.072 0.048	94.4% 95.3% 93.8% 93.5% 95.4%	0.11/- 0.14/- 0.13/- 0.14/- 0.17/-	The cure starting point of the particle-reinforced phenolic resin occurred sooner than that of the neat phenolic resin because nanoparticles catalyze the cure reaction of the phenolic resin at lower temperatures due to microwaves.	[68]
Resole-type phenolic resin	Air bubbles	Chopped glass fiber, ethanol, PTSA catalyst	1000	60	Resin preparation and air bubbles entrapping Acid catalyst mixing Foaming and curing	Phenolic foam with 12% ethanol and 3 or 6 wt% catalyst	0.035		0.148/0.039	Highly uniform phenolic foam was fabricated with 12 wt% ethanol using closed mold microwave foaming process.	[47]
BA	SS		900	240	Missing of BA and SS Microwave foaming in Teflon mold Removal of moisture content	BA:SS(4:6) BA:SS(5:5) BA:SS(6:4) BA:SS(7:3)	0.61 0.64 1.1 1.3	72.64% 71.3% 50.67% 41.7%	3.55/0.075 3/0.07 6.23/0.09 3.67/0.091	Microwave heating initiates the cross-linking of silicates groups, which form an impermeable skin and leads to a highly porous scaffold during foaming.	[60]
TPUR	ADC	CB	500	180 (4 cycles)	Extrusion with blowing agent and CB Microwave foaming	TPUR + ADC TPUR + ADC + CB	0.564 0.050	- - -		The presence of CB in TPUR showed effective microwave heating due to an increase in microwave absorbance, which gave more fine particles. Carbon black additive improves the cell structure and, increases the apparent density but significantly worsens its mechanical properties.	[69]
EPS	Ethanol, hydrogen peroxide, ethanol/water	-	950	180	Injection of solvents in EPS beads Microwave foaming	-	-	-	-/0.029	The better temperature distribution was achieved with hydrogen peroxides using microwave heating.	[43]
Epoxy resin and EPS		Hardener	950	-	Mixing of epoxy resin hardener and EPS beads Microwave foaming Curing	EPS–Epoxy 5% (*w*/*w*) EPS–Epoxy 45% (*w*/*w*)	0.84 0.28	-		The microwave foaming process successfully molded the syntactic foam with sophisticated geometry and smooth surfaces.	[70]
Unexpanded EPS microspheres	Pentane	Phenolic resin	1000		Mixing of EPS, pentane, and phenolic resin Microwave foaming Post curing	Neat EPS foam Composite EPS foam	0.043 0.093		3.35/- 5.80/-	Effective expansion of EPS-syntactic foam at high EPS loading via microwave heating Improved fire-resistant properties due to the formation of a honeycomb structure of composite foam compared to neat polystyrene foam.	[44]
EPDM, PP	ADC	Urea, paraformaldehyde, iron oxide	900	720	The blending of all materials preparation of microcapsules microwave irradiation	EPDM/PP	0.61	-		The microwave technique allows the production of EPDM/PP foam with uniform voids and greater cell sizes of 435 microns, which is almost double produced using the conventional technique.	[61]
CS, PEGDA	Pluronic	-	800–1000	45–240	Mixing of materials and foaming agent Microwave heating	30P70CS1.5 40P60CS1.5		78.90 60.76		Microwave heating allows a homogenous heating process and effectively produced a highly porous interconnected scaffold.	[71]
Titanium and aluminum		Boron carbide (B_4_C)	200–450		A blending of aluminum, titanium and B4C uniaxial pressing to make a cylindrical precursor microwave heating	Al_3_Ti Al_3_Ti + 10% B_4_C Al_3_Ti + 10% B_4_C	-	40% 60% 61%		Microwave heating succeeded in ignition combustion synthesis reaction to produce Al3Ti foam.	[72]
Nickel nitrate	-	Glycine	1000	60	Mixing glycine with a nitrate solution Microwave foaming	Nickel foam	-	40%		Microwave showed high potential to provide a homogenous and increased impregnation rate in the porous scaffold with no surface structure damage. The nanoparticle–microwave interaction caused interconnected nanoparticles, resulting in a percolating network inside the scaffold.	[73]
Graphitic carbon foam (70% porosity)		Boric acid and urea	400	300–18,00	Microwave heating of carbon foam in the presence of additives Vacuum drying Annealing at 500–1100 °C	Boron carbon nitride foam				Microwave treatment effectively activated surface chemical reactions between carbon foam, boric acid, and urea.	[74]
Sucrose	Water	Silica gel	850	180	Mixing of sucrose, silica gel, and water Microwave irradiation thermal treatment at 800 °C under nitrogen atmosphere	Carbon foam	0.17	97% (5–6 nm)		Highly porous carbon/silica foam produced using microwave without any blowing agent.	[59]
PCL	DCM	BPO	700	240	Dissolve PCL in DCM with BPO and mix to homogenous Microwave foaming	PCL-10 wt% BPOPCL-15 wt%BPO	0.40 0.37	63.55% 66.39%		The microwave heating considerably increased the actuation efficiency compared to other heating methods.	[13]
Mimosa tannin extract	Water, furfuryl alcohol, methanol diethylether	-	600	120	Mixing of all solvent and tannin extract Microwave foaming	tannin-furanic foams	0.11		0.44/-	The microwave foaming method of tannic-furanic foam allows a substantially reduced hardening rate of the polymer and the solvent’s blowing. This facilitates faster polymerization and water evaporation and decreased the blowing agent consumption.	[75]

**Table 3:** The possible value proposition of microwave foaming applications.

	Opportunity	Challenge
Environmental	Microwaves can selectively heat water (high dielectric loss) within the matrix, allowing use as a sustainable blowing agent.	Stable addition of water into a polymer matrix
Product quality	Produce controlled pore structures enabled by instant control of heating Higher expansion volume; more product per unit of precursor	Electromagnetic design of homogeneous electric field distribution at large scale
Distributed manufacture based on different (waste-derived) feedstocks/precursors, e.g., packaging	Microwave technologies scalable/can fit in a container enabling small scale local/mobile processing	Understanding sensitive of systems to feedstock variation and incorporating into the electromagnetic design of a system
Reduced processing time and energy consumption	Microwaves apply rapid selective and volumetric heating.	Understanding whether reduced OPEX can offset increased CAPEX for microwave systems
New composites, e.g., polymer syntactic foams	Expansion of higher dielectric loss beads within a microwave transparent matrix	Controlled expansion and hardening/curing of materials

## Abbreviations

EPS	Expanded polystyrene
EPE	expanded polyethylene
EPU	Expanded Polyurethane
PLA	Poly(lactic acid)
MC	Moisture content
AC	Activated carbon
MWCNT	Multi-wall carbon nanotubes
EI	Expansion index
PTSA	p-toluenesulfonic acid
BA	Bottom ash
ADC	Azodicarbonamide
TPUR	Thermoplastic polyurethanes
CB	Carbon black
OPEX	Operating expense
CAPEX	Capital expense
PVOH	Polyvinyl alcohol
Sodium chloride	NaCl
Calcium chloride	CaCl_2_
BIH	Hydrocerol
MC	Moisture content
SS	Sodium silicate
PEGDA	Polyethylene glycol diacrylate
CS	Chitosan
Ti	Titanium
Al	Aluminum
BC	Boron carbide
PCL	Poly(caprolactone)
DCM	Dichloromethane
BPO	Benzoyl peroxide
PTFE	Polytetrafluoroethylene
NiCr	Nickel-chromium
SiC	Silicon carbide
RH	Relative humidity
EPDM	Ethylene-propylene-diene
PP	Polypropylene
